# Can we reduce oxidative stress with liver transplantation?

**DOI:** 10.5937/jomb0-29983

**Published:** 2021-09-03

**Authors:** Mesut Aydin, Yaren Dirik, Canan Demir, Harun Egemen Tolunay, Halit Demir

**Affiliations:** 1 Van Yuzuncu Yil University, Medical Faculty, Department of Gastroenterology, Van, Turkey; 2 Van Yuzuncu Yil University, Vocational School of Healthcare, Van, Turkey; 3 Etlik Zübeyde Hanım Maternity and Women's Health Teaching and Research Hospital, Ankara, Turkey; 4 Van Yuzuncu Yil University, Faculty of Science, Department of Chemistry, Van, Turkey

**Keywords:** oxidative stress, cirrhosis, liver transplantation, oksidativni stres, ciroza, transplantacija jetre

## Abstract

**Background:**

The aim of this study was to determine the levels of lipid peroxidation (MDA) and antioxidants such as reduced glutathione (GSH), catalase (CAT) and superoxide dismutase (SOD) in the blood serum of patients with cirrhosis and liver transplantation.

**Methods:**

In this study, serum malondialdehyde acid (MDA) levels, superoxide dismutase (SOD), reduced glutathione (GSH), and catalase (CAT) activities were measured spectrophotometrically and compared to the results of the healthy control group.

**Results:**

SOD, CAT and GSH activities were significantly decreased in the patient groups compared to the healthy control group (p<0.05). MDA levels were significantly higher in the patient group compared to the healthy control group (p <0.05).

**Conclusions:**

In conclusion, this study demonstrated that oxidative stress may play an important role in the development of liver cirrhosis and in liver transplantation. This study is the first one to show how MDA, SOD, CAT and GSH levels change in liver cirrhosis and liver transplantation, while further studies are essential to investigate antioxidant enzymes and oxidative stress status in patients with cirrhosis and liver transplantation.

## Abstract (sr)

**Uvod:**

Cilj ovog rada je bio da se odrede nivoi lipidne peroksidacije (MDA) i antioksidanti kao što su redukovani glutation (GSH), katalaza (CAT) i superoksid dismutaza (SOD) u krvi seruma pacijenata sa cirozom i transplantacijom jetre.

**Metode:**

U ovom izučavanju nivoi serumskog malondialdehida (MDA), superoksid dismutaze (SOD), redukovanog glutationa (GSH) i aktivnost katalaze (CAT) mereni su spektrofotometrijski i pore|eni sa rezultatima dobijenim kod zdrave kontrolne grupe.

**Rezultati:**

Vrednosti SOD, CAT i GSH bile su značajno niže u grupi zdravih (p < 0,05). Nivoi MDA bili su značajno viši u grupi pacijenata u odnosu na grupu zdravih osoba (p < 0,05).

**Zaključak:**

Na osnovu ovog izučavanja može se zaključiti da oksidativni stres ima značajnu ulogu u razvoju ciroze i transplantacije jetre. Ovo je prvo izučavanje koje ukazuje kako se nivoi MDA, SOD, CAT i GSH menjaju u cirozi jetre i transplantaciji jetre. Sledeća izučavanja biće značajna za proučavanje antioksidativnih enzima i statusa oksidativnog stresa u pacijenata sa cirozom i transplantacijom jetre.

## Introduction

Cirrhosis is a chronic hepatic disease with multiple etiologies and it is characterized by parenchymal damage, fibrosis and nodule formation as well as destruction of lobular and vascular architecture [1]. Diabetes mellitus, non-alcoholic steatohepatitis (NASH), hepatitis B and C, male gender and older age were defined as the risk factors for cirrhosis [2]. Cirrhosis can result in portal hypertension and hepatic dysfunction. Both of these either alone or in combination can lead to many complications, including ascites, varices, hepatic encephalopathy, hepatocellular carcinoma, hepatopulmonary syndrome, and coagulation disorders [3]. Today, liver transplantation is universally accepted as the only treatment option for end-stage liver disease, acute fulminant hepatic failure, hepatocellular carcinoma, hilar cholangiocarcinoma and several metabolic disorders [4]. In addition to surgical risks, liver transplantation may be associated with several problems, including posttransplant immunosuppressive treatment and its side effects, psychological problems associated with liver transplantation, redevelopment of the hepatic disease, and neurological complications [5].

Free radicals are chemical molecules containing an unpaired electron that is generally very reactive. They are continuously formed as by-products of metabolism. The reactive free radicals formed in cells may lead to cell death and tissue damage by oxidizing biomolecules [6]. Free radicals oxidize catalyzed unsaturated fatty acids in membranes through a process called lipid peroxidation, Malondialdehyde (MDA) is a marker of oxidative stress and one of the end-products of lipid peroxidation. MDA level reflects the degree of lipid peroxidation. An increase in free radicals leads to overproduction of MDA [7] [8]. Lipids, proteins, carbohydrates and other cell components undergo oxidation, leading to significant damage to cellular structures. The accumulation of this damage is called oxidative stress [9]. Kupffer cells exposed to harmful reactions are the main effectors responsible for the formation of reactive oxygen species (ROS) that affect hepatic stellate cells (HSC) and hepatocytes. ROS-activated HSC are exposed to a phenotypic stimulation and deposit excessive amounts of extracellular matrix, altering the normal architecture of the liver and negatively affecting hepatic function [10]. In addition, ROS stimulate necrosis and apoptosis of hepatocytes, leading to hepatic damage and progression to end-stage hepatic disease [11]. In patients with hepatic cirrhosis, stress-activated macrophages and neutrophils release high concentrations of oxidants leading to oxidation. This, in turn, may damage DNA, proteins and carbohydrates. Damages mediated by lipid peroxidation caused by toxic agents are known to result in hepatic injuries ranging from inflammation to necrosis in case of continuous exposure to the agent and insufficiency of antioxidant systems [12] [13]. Oxidative stress-induced cell damage may lead to fibrosis and cirrhosis in the liver [14].

The term »antioxidant« describes the molecules that can stabilize or inactivate free radicals before they damage cells [15]. Some antioxidant enzymes include superoxide dismutase, catalase and glutathione reductase.

Superoxide dismutase (SOD) is known to exist in all aerobic cells and plays a role in the defense against superoxide radicals formed as a result of aerobic reactions [16]. In biological systems, SOD enzyme accelerates the reaction by approximately four orders. SOD enzyme works together with H_2_O_2_-eliminating enzymes such as catalase, and glutathione reductase [17].

Catalase (CAT) enzyme occurs in peroxisomes and plays role in the conversion of hydrogen peroxide to water and oxygen [18].

Reduced glutathione (GSH) plays a very important role in the elimination of harmful reactive oxygen radicals and maintenance of enzymatic activities. It catalyzes the reduction of oxidized glutathione to glutathione. GSH, one of the most important intracellular antioxidant molecules, has also many physiological functions such as detoxification of xenobiotics, amino acid transport, maintaining the reduced form of sulfhydryl groups, and coenzyme role in some enzymatic reactions in addition to its role in antioxidant defense system [19].

The aim of this study was to determinate level of oxidative stress and antioxidative defense parameters in patients with hepatic cirrhosis and patients undergoing liver transplantation and compared that to healthy controls patients.

## Materials and Methods

### Patients

The results obtained in the present study were from a total number of 94 subjects out of which 31 patients with hepatic cirrhosis, 30 patients undergoing liver transplantation for hepatic cirrhosis and 33 healthy controls cases. 25 of the cases were female and 36 of the cases were male. The control group was 19 men and 14 women. There was no difference in age, gender, co-morbidities, between subjects. The demographic, clinical and biochemical data of both groups were recorded and comparatively analyzed. The patients provided 4 mL venous blood samples from the brachial vein collected into biochemistry tubes and the sera obtained by centrifuging the samples at 5000 rpm for 5 minutes. Sera were stored at -20°C until the time of analysis. In these samples after thawing were spectrophotometrically measurements activity of SOD, CAT, GSH and MDA levels in clinical-biochemical laboratory.

All patients and volunteers provided written informed consent. The study was approved by the Ethics Committee of Batman Regional State Hospital on June 13, 2018 with the approval number of 2018/98-103-109.

### Biochemical Measurements

SOD activity was determined by using the proposed method of Popov [20]. CAT enzyme activity was determined using the method described by Aebi [21]. GSH level using the method described by Beutler [22]. MDA level was determined according to the method reported by Gutteridge [23].

### Statistical Analysis

Descriptive statistics for the features under consideration were expressed as mean and standard deviation. The Kolmogorov-Smirnov test was used to test the normality of the distribution of data. One-way analysis of variance (ANOVA) was used in cases with normal distribution. Kruskal-Wallis test was used in cases where normal distribution was not evident. The statistical significance level was set at p<0.05 and the SPSS statistical software, version 19.0 (SPSS Inc, Chicago, III, USA) was used for analyses.

## Results

**Figure 1:**
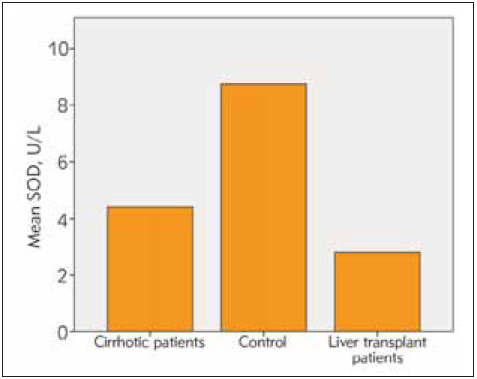
SOD activities in examined groups

**Figure 2:**
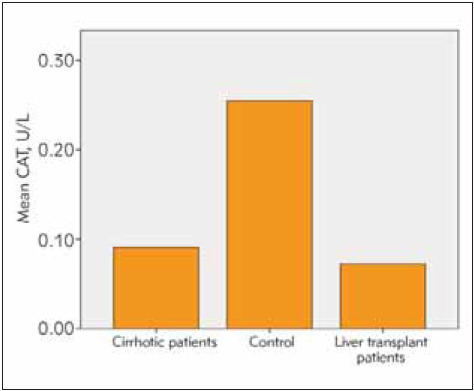
CAT activities in all groups

**Figure 3:**
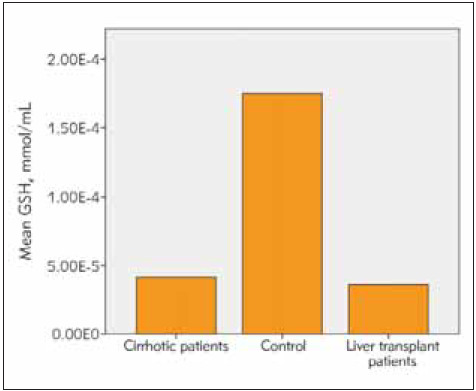
GSH levels in examined groups

**Figure 4:**
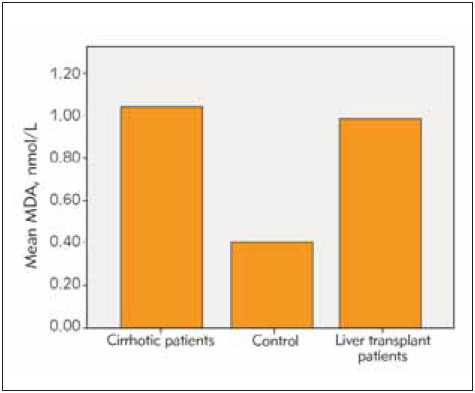
MDA levels in examined groups

Among the patients undergoing liver transplantation, 25 had hepatitis B and the remaining 5 patients had hepatic cirrhosis due to NASH as reasons for transplantation. The time since liver transplantation was over 1 year in all patients. All patients received transplants from live donors and all donors were blood group-matched relatives. 39 (41%) of the cases were female and 55 (59%) of them were male. The age range of the included patients was 18-75 years (mean age 48 years). The mean age in the cirrhosis group is 45, in the transplant group 51 and in healthy group was 47. Cirrhotic patients had an average diagnosis of 7 years. In the group of patients with liver transplantation, at least 2 years and at most 8 years had passed since the liver transplantation. All of the liver transplant patients were using tacrolimus and mycophenolate mofetil as immunosuppressive drugs. Eighteen of the liver transplant patients were using hepatitis b immunoglobulin according to the anti-HBs titer. The average amount of smoking in cirrhotic patients was 16 pack-years. The patients had a mean duration of tobacco use of 12 pack-years and no patients used tobacco after transplantation. The three patients with hepatic cirrhosis had a mean tobacco use of half packs per day. Smoking rate in healthy volunteers was 13 pack-years. The mean AST, ALT, Bilirubin and thrombocyte values were within normal limits in the liver transplant patients and the healthy group. In the cirrhotic group, AST ALT values were within normal limits, but AST value was greater than ALT, consistent with cirrhosis and mean INR value was 2.1 while mean platelet count was 73.000/mm^3^. Descriptive statistics and comparison results for SOD, CAT, GSH and MDA are given in Table 1. The dif ference between the group averages was found to be statistically significant (p<0.05) (Table 1). Accordingly, activities of SOD, CAT and GSH levels were significantly lower in patients with cirrhotic and liver transplantation compared to the control group, while the MDA level was found to be quite high (p<0.05). Although SOD, CAT, GSH and MDA levels were lower in transplant patients compared to cirrhotic patients, this difference was not statistically significant (p>0.05) (Figure 1, Figure 2, Figure 3, Figure 4).

**Table 1:** The comparison results between biochemical oxidative and antioxidative parameters value in tested subjects Different letters represent different group means (p<0.05)

	Cirrhotic (n=31) Mean±SD	Control (n=33) Mean±SD	Liver transplant (n=30) Mean±SD	p
SOD (U/L)	4.4133±0.63409	8.7506±0.33384	2.8127±0.83411	p<0.05
CAT (U/L)	0.0906±0.00868	0.2542±0.01967	0.0724±0.01870	p<0.05
GSH (mmol/mL)	0.00004±0.00002	0.0002±0.00001	0.00003±0.00002	p<0.05
MDA (nmol/L)	1.0405±0.19701	0.3827±0.04341	0.9827±0.15931	p<0.05

## Discussion

Cirrhosis is a progressive disease characterized by parenchymal tissue loss, diffuse increase in connective tissue, formation of regeneration nodules and destruction of vascular architecture [24]. Liver transplantation has become the definitive treatment option in end-stage liver disease patients or patients with an unacceptably poor quality of life.

Reactive oxygen radicals have been associated with many diseases including autoimmune diseases like rheumatoid arthritis, diabetes mellitus, atherosclerosis, obesity, hypertension and cardiovascular diseases such as ischemia [25] [26]. Studies report that oxidative stress has a significant impact on the development of many types of cancer including renal, lung, liver and breast cancers [27] [28]. High levels of reactive oxygen species cause inflammation leading to hepatic fibrosis over time and antioxidants are highly effective in the prevention of damage due to oxidative stress caused by free radicals [29]. The level of oxidative stress increases with the stage of cirrhotic disease. Dede et al. [30] in their study found that the level of malondialdehyde, an indicator of oxidative stress, increases in proportion with the Child's stage in patients with hepatic cirrhosis. Galicia-Moreno et al. [31] in their study suggested that MDA levels increase with the stage of cirrhosis and abstinence from alcohol decreases MDA levels and may stop the progression of the disease in patients with alcoholic cirrhosis. Pratico et al. [32] in their study reported that lipid peroxidation is increased in cirrhotic patients. Other studies showed that oxygen radicals and lipid peroxidation are associated with an increase in collagen synthesis and development of fibrosis [33] [34]. It has been reported in various studies that the increase in leptin stimulates liver fibrosis. This stimulus is known to increase the release of TGF-Beta via the hepatic stellate cell and this causes fibrosis [35].

Our study found that malondialdehyde level, another indicator of oxidative stress, is increased in cirrhotic and liver transplant patients compared to normal population. Oxidative stress was demonstrated to play a role in the development of hepatic damage in many studies [36]. Interestingly, we found our study that levels of these antioxidant enzymes were still significantly lower after liver transplantation compared to normal population. This shows that the oxidative processes, which can cause hepatic damage severe enough to require transplantation, persist after transplantation. Again, the finding that levels of malondialdehyde, one of the end-products of lipid per oxidation, are higher both during cirrhosis and after transplantation compared to healthy individuals is another indicator of ongoing cell damage. This probably shows that oxidative stress that leads to cirrhosis and subsequently to liver transplantation, also persists after transplantation. Thus, liver transplantation is not able to save the patient from oxidative stress but only provides the patient with a new liver that can be damaged by oxidative stress. Hence, it is clear that the liver should be protected from oxidative stress. Immunosuppressive drugs can also alter the oxidative stress state. The decrease in non-significant oxidative stress markers after transplantation may be due to the use of immunosuppressive drugs. Further studies are needed to determine this. Therefore, it may be thought that addition of antioxidants to the treatment of patients who underwent liver transplantation will slow down hepatic damage and prolong liver transplant survival. A study proved that L-theanine, an amino acid found in green tea, has the ability to prevent alcoholic hepatic injury by increasing the antioxidant reserve. L-theanine significantly inhibits the ethanol-induced increase in ALT, AST and MDA and the decrease in antioxidant enzyme activities, including SOD and CAT activities as well as GSH levels. Moreover, vitamin E, with its antioxidant properties, is believed to be beneficial in preventing diseases associated with oxidative stress. It has been proven that vitamin E is able to reverse redox states, prevent oxidative stress and decrease apoptosis and can be used as a therapeutic agent against ethanol-induced hepatic oxidative damage. In addition, raxofelast, a vitamin E analog, has the ability to prevent lipid peroxidation in mice exposed to ethanol [37] [38] [39]. Nacetyl cystein, a reactive oxygen scavenger, is effective in the treatment of alcoholic hepatic damage [40]. Augusto et al. in their study including 20 cirrhotic patients and 20 healthy subjects performed in 2014, found that there was no significant difference in MDA levels between the control and cirrhotic groups while MDA level was lower in liver transplant patients, compared to the healthy group [41]. In our study, we found that MDA levels were high in both liver transplant and cirrhotic patients compared to the control group. This shows us that hepatitis B and NASH, which are the cause of cirrhosis in our patients, are not treated with liver transplantation, cirrhosis caused by them is treated and the oxidative process continues. Alcohol was found to be significantly effective on non-enzymatic substances such as glutathione, vitamin A, C and E and enzymatic systems such as catalase and superoxide dismutase, which play a role in the protection of cells against repetitive free radical attacks and studies in alcohol-administered animals demonstrated that lipid peroxidation in serum (malondialdehyde acid) was increased and conversely, there was a significant decrease in vitamin E and C concentrations, which are important markers of antioxidants [42] [43] [44]. In conclusion, the cyclic variations in oxygen saturation may increase ROS production in cirrhotic patients. This may increase oxidative stress [9]. The present study also confirms this and, likewise, liver transplant patients also have a similar condition. Therefore, it is observed that there is an increased level of cellular damage in both cirrhotic and liver transplantation patients due to increased oxidative stress.

SOD is a very important antioxidant enzyme. SOD protects the cells against many radicals. The very potent enzymatic activity of SOD is reported to be decreased by ionizing radiation. SOD activity was significantly lower in both cirrhotic and liver transplantation patients compared to the control group (p<0.05). The decreased SOD activity found in this study can be explained by the inability of SOD to exert its function as a part of the detoxification process due the effect of ROS. Likewise, decreased SOD activity may be due to the inhibitory effect of hydrogen peroxide. CAT is a very potent antioxidant enzyme. CAT protects the organism against free radicals such as hydrogen peroxide, superoxide anion, and hydroxyl radicals [45]. CAT enzyme activity was found to be significantly lower in both cirrhotic and liver transplantation patients compared to the control group (p<0.05). This decrease in CAT activity in hepatic cirrhosis patients may be due to the inactivation of catalase by H_2_O_2_. This may explain the decreased catalase activity in the sera of hepatic cirrhosis patients.

The conversion of H_2_O_2_ to H_2_O and O_2_ may be a reason for the decreased CAT activity. In conclusion, CAT provides protection against the harmful effects of hydrogen peroxide.

Glutathione is a tripeptide formed by glutamic acid, cysteine and glycine. GSH level was found to be significantly lower in both cirrhotic and liver transplantation patients compared to the control group (p<0.05). In conclusion, the reduced GSH levels in cirrhotic patients can increase cellular damage. In this case, GSH level is also observed to decrease due to increased oxidative damage in hepatic cells.

## Conclusion

According to our results study demonstrate increased oxidative stress damage and decreased antioxidant CAT and SOD activities and GSH levels in both cirrhotic and liver transplantation patients. Currently, there are very few studies focusing on longterm follow-up of surviving liver transplant recipients and the present study may therefore explain the uncertainties about the long-term outcomes and provide new insights into cardiovascular outcomes, hepatic functions and damage, endothelial dys function and aging in this group of patients. Our study found that superoxide dismutase and catalase activities and reduced glutathione levels were significantly lower, while malondialdehyde acid level, which is another marker of increased oxidative stress, was significantly increased in cirrhotic patients compared to normal population. Therefore, although several studies described oxidative stress in hepatic cirrhosis and recommended the use of supportive antioxidant treatment at this stage, antioxidant treatment may also be considered in liver transplantation patients since oxidative stress persists at a significant level after liver transplantation as we have described in our study.

In conclusion, oxidative stress may play a critical role in the development of hepatic cirrhosis and of damage to the transplanted liver. This the first study demonstrating the roles of MDA, SOD, CAT, and GSH in hepatic cirrhosis and liver transplantation. There fore, further studies in liver transplantation patients are required for indications of antioxidative therapy, in prevention and diagnostics of liver dam-age. According to the available measured data, liver transplantation patients is closely associated with changes in the redox status. It is necessary to minimize the oxidative stress and continue, according to the literatüre.

## Conflict of interest statement

All the authors declare that they have no conflict of interest in this work.
